# Species-Specific Enhancer Activity of OCT4 in Porcine Pluripotency: The Porcine *OCT4* Reporter System Could Monitor Pluripotency in Porcine Embryo Development and Embryonic Stem Cells

**DOI:** 10.1155/2022/6337532

**Published:** 2022-06-11

**Authors:** Seung-Hun Kim, Mingyun Lee, Kwang-Hwan Choi, Jinsol Jeong, Dong-Kyung Lee, Jong-Nam Oh, Gyung Cheol Choe, Chang-Kyu Lee

**Affiliations:** ^1^Department of Agricultural Biotechnology, Animal Biotechnology Major, And Research Institute of Agriculture and Life Science, Seoul National University, Seoul 08826, Republic of Korea; ^2^Research and Development Center, Space F Corporation, Hwasung, Gyeonggi-do 18471, Republic of Korea; ^3^Designed Animal & Transplantation Research Institute, Institute of Green-Bio Science and Technology, Seoul National University, Gangwon-do 25354, Republic of Korea

## Abstract

The present study examined the activity and function of the pig *OCT4* enhancer in the porcine early embryonic development stage and porcine authentic embryonic stem cells. OCT4 is known as a pluripotent regulator, and its upstream regulatory region-based dual-fluorescence protein reporter system controlled by distal and proximal enhancers is broadly used in studies examining the states and mechanism of pluripotency. We analyzed how this reporter system functions during early embryo development and in stem cells using a previously established porcine-specific reporter system. We demonstrated that the porcine *OCT4* distal enhancer and proximal enhancer were activated with different expression patterns simultaneously as the expression of pluripotent marker genes changed during the development of in vitro pathenotes and the establishment of porcine embryonic stem cells (ESCs). This work demonstrates the applicability of the porcine *OCT4* upstream region-derived dual-fluorescence reporter system, which may be applied to investigations of species-specific pluripotency in porcine-origin cells. These reporter systems may be useful tools for studies of porcine-specific pluripotency, early embryo development, and embryonic stem cells.

## 1. Introduction

The characteristics of pluripotent stem cells are proliferation and differentiation, which may be useful tools for therapeutic research, such as regenerative medicine. Pluripotent stem cells (PSCs) have two different states of pluripotency. Naïve or primed PSCs are related to the activation of LIF or FGF signaling, respectively. The mouse is a well-researched model for naïve and primed pluripotent states, which have a clear distinction. The reference cells of pluripotent states are present according to the level of pluripotency, such as mouse embryonic stem cells (naïve), mouse epiblast stem cells, and embryonic carcinoma cells (primed) [1–4]. Various studies have also reported porcine pluripotent stem cells [5–7]. A recent remarkable achievement was made in pigs with the establishment of authentic embryonic stem cells [8]. However, only primed pluripotent stem cells of pigs have been reported [8, 9]. One key difference between naïve and primed states in pluripotent stem cells is the expression of *OCT4*.

The pluripotent marker *OCT4* is one of many pluripotency-related genes that has been studied as a reporter gene because it is only expressed in pluripotent cells [10]. The transcription factor *OCT4* is an important marker of the undifferentiated status in early mammalian embryonic development and embryonic stem cells. It plays a critical role as a central regulator in maintaining pluripotency and self-renewal. *OCT4* contains a core promoter and two conserved enhancers, the distal enhancer (DE) and proximal enhancer (PE) [11–13]. Enhancers have multiple cognate binding sites where various transcription factors may be attached to regulate the expression of genes. Some sites are close to the promoter, and other sites are far away. The former sites are called proximal enhancers (PEs), and the latter sites are called distal enhancers (DEs) [14]. These two types of enhancers are used to produce *OCT4* in various pluripotent cells and work simultaneously or sequentially. Because different factors are present depending on the pluripotent state, *OCT4* has two enhancers to form OCT4 in different environments and it is configured to operate in two different environments. One study of a mouse *Oct4* upstream region model revealed that the two enhancer regions were activated differently. DE is a key element of the *Oct4* gene in naïve pluripotent cells, such as mouse embryonic stem cells, germ cells, and inner cell mass [11, 15], and *Oct4* PE is specifically primed in pluripotent cells, such as mouse epiblasts and embryonic carcinomas [11, 13].

Pluripotent stem cells exhibit heterogeneity during culture [16–18]. Therefore, an *OCT4* upstream-based dual-reporter system may be used to classify the states of pluripotency in mixed populations of pluripotent cells and separate more naïve cells and more primed cells in these mixed populations. Although it is one of the most necessary factors in the study of stem cells and pluripotency, the function of a porcine-specific *OCT4* reporter system in porcine-origin cells has not been reported. Therefore, it is necessary to compare and confirm the activity of the *OCT4* enhancers with the acquisition of pluripotency in the reprogramming process. Many studies have been performed using species-specific reporter systems in other species for introduction into cells derived from different species. The mouse *Oct4* reporter system is well developed and has been applied for pluripotency studies [12, 19]. The h*OCT4*-*Δ*PE-GFP reporter system demonstrated problems in humans, such as slight activity in primed human pluripotent stem cells, but it could be used to distinguish naïve from primed cells [20]. Although research on porcine pluripotent stem cells is important for human therapeutic research [21], many researchers have tried to establish naïve pluripotent stem cells and authentic-induced pluripotent stem cells [8, 9, 22–25]. However, they have not been reported and one of the reasons is the lack of research on useful tools for studying species-specific pluripotency, including a porcine *OCT4* upstream-based reporter system, compared to other species. Many researchers have sought to develop a porcine-specific reporter system given its importance.

Recently, the study of pluripotency has taken a species-specific approach. Differences in pluripotency-related gene expression and mechanisms between species have been elucidated. This is also the case with OCT4. First, the expression of OCT4 in murine blastocysts is limited to the ICM, whereas in porcine and human blastocysts, it is expressed in both the ICM and TE [26]. In particular, knockdown of OCT4 with siRNA affects TE development in pigs [27]. In addition, OCT4 loss in the early stage of embryos showed different results in mice and humans [28], so its role differs. Furthermore, the enhancer of OCT4 also has species-specific features. An m*Oct4-* and h*OCT4*-based reporter was introduced in porcine pluripotent cells, but it is not a porcine-specific reporter system [29]. A porcine *OCT4* upstream region-based eGFP reporter system was introduced in porcine embryonic fibroblasts and functioned after SCNT and reprogramming, but the activities of the distal enhancer and proximal enhancer were not separated [30]. A porcine *OCT4* enhancer-based dual-reporter system was active in mouse pluripotent stem cells, but a luciferase assay and analysis of porcine pluripotent cells with a reporter system were not performed [31]. Our previous study analyzed the *OCT4* upstream regions. The results showed that the sequence and function of distal and proximal enhancers of porcine *OCT4* were similar to those of other mammals. However, a large difference between species was found in the *Oct4* upstream region nucleotide sequence and the differences in expression occurred when the *Oct4* upstream region-based reporter systems constructed from one species were inserted into another species. Porcine proximal enhancer-based vectors did not work properly in mouse pluripotent stem cells [32]. Therefore, the porcine-specific *OCT4* reporter system, which is important for porcine pluripotency research, required functional evaluation in porcine-origin pluripotent cells. We needed to establish a reference by checking the activity and changes in the porcine *OCT4* enhancers according to the state of pluripotency during the process of reprogramming. Therefore, we performed functional tests of the porcine *OCT4* reporter system in porcine-induced pluripotent stem cells in this study.

## 2. Materials and Methods

### 2.1. Animal Welfare

The authors assert that all procedures in this work complied with the ethical standards of the relevant national and institutional guides on the care and use of laboratory animals. The Institutional Animal Care and Use Committee, Seoul National University, approved the care and experimental use of pigs (SNU-181024-8, SNU-191025-4-1, and SNU-201019-1-1). The ovaries used in the present study were donated from local slaughterhouses (Dodram, Korea; Samsung, Korea) only for research. Pregnant ICR mice were purchased from SAMTACO BIO Inc., Korea. The mice were cared for according to the standard protocol of the Institute of Laboratory Animal Resources and sacrificed by cervical dislocation after anesthesia.

### 2.2. Porcine *OCT4* Upstream Region-Derived Dual-Reporter System

Porcine-specific OCT4 upstream region-derived dual-reporter systems were produced as described previously [32]. The following two vectors were used primarily in this study: p*OCT4*-*∆*PE-eGFP vector (DE-GFP) containing conserved regions (CRs) 1 and 4 and a p*OCT4*-∆DE-DsRed2 vector (PE-RFP) containing CR1, 2, and 3. For analysis of the expression pattern of distal and proximal enhancers using two previously constructed reporter systems, an identical fluorescent (eGFP) vector was used. The p*OCT4*-∆PE-eGFP vector (DE-GFP) and p*OCT4*-∆DE-eGFP vector (PE-GFP), which contained CR1, 2, and 3 in the eGFP vector, were transfected into porcine embryonic fibroblasts cultured under LIF- or FGF-reprogramming conditions. All vectors were linearized by AseI and Hind III before use to remove the CMV promoter and for cell transfection.

### 2.3. In Vitro Embryo Production

The ovaries of prepubertal gilts were obtained from a local slaughterhouse (Anyang-si, Gyeonggi-do, Korea) and transferred to the laboratory in warm saline. Cumulus-oocyte complexes (COCs) were collected by aspirating 3 to 7 mm follicles of the prepubertal gilts using a 10 mL syringe with an 18-gauge needle. Sediments were washed with TL–HEPES–PVA medium, and oocytes with compact cumulus cells and granulated cytoplasm were selected for in vitro maturation. The washed COCs were cultured in tissue culture medium (TCM-199; Life Technologies, Carlsbad, CA, USA) containing 10 ng/mL epidermal growth factor, 1 mg/mL insulin, and 10% porcine follicular fluid for 44 hours at 39°C at 5% CO_2_ and 100% humidity. The COCs were matured with 10 IU/mL gonadotropin hormone, pregnant mare serum gonadotropin (Lee Biosolutions, Maryland Heights, MO, USA), and human chorionic gonadotropin for the first 22 hours. The COCs were then matured under hormone-free conditions. To generate parthenotes, cumulus-free oocytes were activated with an electric pulse (1.0 kV/cm for 60 ms) in activation medium (280 mM mannitol, 0.01 mM CaCl_2_, and 0.05 mM MgCl2) using a BTX Electro Cell Manipulator (BTX, CA, USA), followed by 4 hours of incubation in PZM3 medium containing 2 mmol/L 6-dimethylaminopurine.

### 2.4. Cytoplasmic Injection of the DNA-Lipofectamine Complex

For the microinjection assay, 5 *μ*L of the 90 ng/*μ*L reporter system (PE + RFP, DE + GFP) in combination with 1 *μ*L of lipofectamine (Stem reagent; Thermo Fisher Scientific) was incubated for 5 min in Media-199 (Gibco) and the final DNA concentration was 15 ng/*μ*L. One hour after PA, the embryos were injected with 2 pl of plasmid-lipofectamine solution in manipulation medium. The microinjection procedure was conducted using a micromanipulator (Eclipse TE2000, Nikon, Tokyo, Japan) with a Femtotip II microinjector (Eppendorf, Hamburg, Germany). After microinjection, the embryos were washed and then cultured in PZM3 medium for 6 days.

### 2.5. Derivation of Primary Colonies

To optimize pig ESC culture medium, hatched parthenogenetic blastocysts were seeded on feeder cells composed of mitotically inactivated mouse embryonic fibroblasts (MEFs) under various culture conditions. ESC medium consists of basal medium, replacement of fetal bovine serum (FBS), and signaling molecules. Basal medium was composed of KnockOut™ Dulbecco's modified Eagle's medium (KO-DMEM) containing 2 mM GlutaMAX, 0.1 mM *β*-mercaptoethanol, 1x MEM nonessential amino acids, and 1x antibiotic-antimycotic (all from Gibco). The tested serum replacements were 20% KnockOut™ Serum Replacement (KSR) (Gibco), 1x N2/B27 supplements (Gibco), a combination of 20% KSR and 0.2% chemically defined lipid concentrate (LC) (Gibco), and a combination of 5% KSR and 1x N2/B27 supplements. Treated signaling molecules were the 10 ng/mL human recombinant basic fibroblast growth factor (hrFGF2) (R&D Systems), 10 ng/mL activin A (ActA) (R&D Systems), and 1 *μ*M CHIR99021 (CH) (Cayman Chemical, MI, USA). After 7 days, primary colonies of embryonic stem cells were observed and then fixed with 4% paraformaldehyde for further analysis. All cultures were performed under humidified conditions containing 5% CO_2_ and 5% O_2_ at 39°C.

### 2.6. Culture of Pig Embryonic Stem Cells

Established pig ESCs were cultured with ESC medium supplemented with the 10 ng/mL human recombinant basic fibroblast growth factor (FGF2) (R&D Systems), 10 ng/ml activin A (ActA) (R&D Systems), and 1 *μ*M CHIR99021 (CH) (Cayman Chemical). ESC medium consisted of KnockOut™ Dulbecco's modified Eagle's medium (KO-DMEM) containing 20% KnockOut™ Serum Replacement (KSR), 0.2% chemically defined lipid concentrate (LC) (Gibco), 2 mM GlutaMAX, 0.1 mM *β*-mercaptoethanol, 1x MEM nonessential amino acids, and 1x antibiotic-antimycotic (all from Gibco, USA). Pig ESCs were subcultured every 5–7 days. Expanded colonies were detached from the feeder cells and dissociated into small clumps using pulled glass pipettes. These clumps were transferred onto new feeder cells and cultured with 10 *μ*M Y-27632- (Santa Cruz) containing ESC medium for 24 h. After 24 h, attached clumps were cultured with ESC medium in the absence of Y-27632 for 4–6 days.

### 2.7. Reverse Transcription-Polymerase Chain Reaction

Total RNA from individual samples was extracted using TRIzol® reagent (Invitrogen, MA, USA) according to the manufacturer's instructions. Complementary DNA (cDNA) was synthesized using a High-Capacity RNA-to-cDNA Kit (Applied Biosystems) according to the manufacturer's instructions, producing a final volume of 20 *μ*L. Derived cDNA samples were amplified with 2x PCR master mix solution (iNtRON) and 2 pmol primers, as shown in Table S2. PCRs were performed in a thermocycler under the following conditions: 95°C for 5 min, 35 cycles of denaturation at 95°C for 30 s, annealing for 30 s (annealing temperatures depending on each primer set), extension at 72°C for 30 s, and a final extension at 72°C for 7 min. Amplified PCR products were visualized using electrophoresis on a 1% agarose gel stained with ethidium bromide.

### 2.8. Quantitative Real-Time Polymerase Chain Reaction for Embryos

Total RNA from pooled embryos at each stage of in vitro-produced embryos (6–8 cell, *n* = 20; morula, *n* = 10; and blastocysts, *n* = 5) was isolated using an Arcturus® PicoPure® RNA Isolation Kit (Applied Biosystems, Foster City, CA, USA) following the manufacturer's instructions. cDNA was synthesized using a High-Capacity RNA-to-cDNA Kit (Applied Biosystems). The cDNA samples were amplified using a Power SYBR Green Master Mix (Applied Biosystems) containing 1 pmol of each primer set listed in Table S2 in a 10 *μ*L reaction volume. Amplification and detection were conducted using the ABI 7300 Real-Time PCR System (Applied Biosystems) under the following conditions: one cycle of 50°C for 2 min and 95°C for 10 min, followed by 40 cycles of denaturation at 95°C for 15 s and annealing/extension for 1 min (annealing/extension temperatures dependent on each primer set). The dissociation curves were analyzed, and the amplified products were loaded onto gels to confirm the specificity of the PCR products. The relative expression level was calculated by normalizing the threshold cycle (*Ct*) values of each gene to that of the reference gene beta-actin (ACTB) via the delta-delta *Ct* method.

### 2.9. Quantitative Real-Time Polymerase Chain Reaction for Embryonic Stem Cells

Total RNA was extracted using TRIzol® Reagent (Invitrogen), and cDNA was synthesized using the High-Capacity RNA-to-cDNA Kit (Applied Biosystems). Extracted cDNA samples were amplified with a DyNAmo HS SYBR Green qPCR Kit (Thermo Scientific) containing 1–2 pmol of each primer set listed in Table S2 in a 10 *μ*L reaction volume. Amplification and detection were conducted using the ABI 7300 Real-Time PCR system (Applied Biosystems) under the following conditions: one cycle of 50°C for 2 min and 95°C for 10 min, followed by 45 cycles of denaturation at 95°C for 15 sec and annealing/extension for 1 min (annealing/extension temperatures depended on each primer set). The dissociation curves were analyzed, and the amplified products were loaded on gels to confirm the specificity of the PCR products. The relative expression level was calculated by normalizing the threshold cycle (*Ct*) values of each gene to that of *ACTB* via the Δ^–*C*^*^t^* method [33].

### 2.10. Alkaline Phosphatase Staining

Cells were fixed with 4% paraformaldehyde for 30 min. Fixed cells were stained with a solution containing nitro blue tetrazolium chloride (NBT) and 5-bromo-4-chloro-3-indolyl phosphate toluidine salt (BCIP) stock solution (Roche) in a buffer solution for 30 min at room temperature. Stained cells were then examined under an inverted microscope.

### 2.11. Immunocytochemistry for Embryos

Each stage of embryos without zona pellucida was fixed in 4% paraformaldehyde for 15 min at room temperature. Fixed samples were permeabilized using 1% Triton X-100 for 1 hour at room temperature and washed three times with phosphate-buffered saline (PBS). The embryos were blocked using 10% goat serum or donkey serum in PBS for 1 hour at room temperature. Samples were stained with anti-GFP (5 *μ*g/mL), RFP (1 *μ*g/mL), and anti-OCT4 (1 *μ*g/mL) or anti-SOX2 (2.5 *μ*g/mL) in PBS containing 10% goat serum at 4°C overnight (Table S1). After washing 3 times in washing solution (PBS with 0.2% Tween-20 and 1% BSA for 10 min), embryos were incubated with goat anti-mouse Alexa 647 (Invitrogen, Carlsbad, California, USA), goat anti-chicken Alexa488 (Invitrogen), and goat anti-rabbit Alexa555 (Invitrogen) in PBS with 10% goat serum or donkey serum at RT for 1 hour. All samples were washed 3 times with washing solution after secondary antibody treatment. Immunostained embryos were mounted on a glass slide with Prolong Gold and DAPI (Invitrogen) and cured for more than 24 hours. We described the list of antibodies in Table S1. Images of stained cells were captured using a confocal microscope and processed by the ImageJ program.

### 2.12. Immunocytochemistry for Embryonic Stem Cells

Before fixation, cell samples were preincubated for 10 min at 4°C and fixed with 4% paraformaldehyde for 30 min. After washing twice with DPBS (Welgene), samples were treated for 1 h with 10% goat serum in DPBS to prevent nonspecific binding. Serum-treated cells were incubated overnight at 4°C with primary antibodies. The primary antibodies used were as follows: OCT4 (Santa Cruz Biotechnology; 1 : 200), SOX2 (Millipore; 1 : 200), NANOG (PeproTech; 1 : 200), SSEA1 (Millipore; 1 : 200), and SSEA4 (Millipore; 1 : 200). When antibodies against intracellular proteins such as OCT4, SOX2, and NANOG were used, fixed cells were treated for 15 min with 0.1% Triton-X100 (Sigma–Aldrich) before serum blocking. After incubation with the primary antibody, the cells were treated for 3 h at room temperature with Alexa Fluor-conjugated secondary antibodies. Nuclei were stained with Hoechst 33342 (Molecular Probes). Images of stained cells were captured using an LSM 700 laser scanning microscope (Carl Zeiss) and processed with the ZEN 2012 Light Edition program (Carl Zeiss).

### 2.13. Confocal Imaging Process

Confocal immunofluorescence pictures were taken with a Leica SP8X (Leica Microsystem, Wetzlar, Germany) and processed by the Fiji (ImageJ) program.

### 2.14. Karyotyping

Karyotyping of cells using a standard G-banding chromosome and cytogenetic analysis were performed at GenDix Laboratories. Twenty metaphases were analyzed.

### 2.15. Spontaneous Differentiation of Pig ESCs In Vitro Using the Embryoid Body Method

Cultured pig ESCs were dissociated into single cells using TrypLE Express (Gibco) and cultured on ultralow-attachment plates (Sigma-Aldrich) with DMEM containing 15% (*v*/*v*) FBS and 10 *μ*M Y-27632 (day 1 only) without other cytokines for 5 days. After suspension culture, the dissociated cells aggregated and formed EBs, which were seeded on 0.1% (*w*/*v*) gelatin-coated plates and cultured for 2–3 weeks in DMEM containing 15% (*v*/*v*) FBS. The resulting differentiated cells were lysed with TRIzol reagent (Invitrogen) for further analysis.

### 2.16. Mycoplasma Test

Genomic DNA was extracted using the G-Spin Total DNA Extraction Kit (iNtRON Biotechnology Inc.), and the mycoplasma test was performed using the e-Myco™ plus Mycoplasma PCR Detection Kit (iNtRON Biotechnology, Inc.) according to the manufacturer's protocol. Sample control and internal control bands indicate that sample preparation and PCR amplification were appropriately conducted, respectively.

### 2.17. Teratoma Formation Assay

Pig ESCs at 70%–80% confluence were used for transplantation. Approximately 5–10 × 10^6^ ESCs were resuspended in 200 *μ*L of ESC culture medium containing 50% (*v*/*v*) BD Matrigel Matrix (BD Biosciences) and 10 *μ*M Y-27632. Next, the resuspended pig ESCs were injected subcutaneously into 5-week-old athymic nude mice (ORIENTBIO). At 2–3 months after transplantation, 1 to 2 cm teratomas were collected, fixed in 4% (*w*/*v*) paraformaldehyde, embedded in paraffin, and stained with H&E for light microscopic examination.

For genotyping of teratomas, genomic DNA was extracted from teratomas and the peritoneum of athymic nude mice using the G-spin Total DNA Extraction Kit (iNtRON Biotechnology). Genomic DNA samples were amplified using 10 pmol of species-specific primers and 2x PCR master mix solution (iNtRON Biotechnology). PCRs were performed in a thermocycler under the following conditions: 95°C for 5 min; followed by 25 cycles of denaturation at 95°C for 30 s, annealing at 60°C for 30 s, and extension at 72°C for 30 s and a final extension at 72°C for 7 min. Amplified PCR products were electrophoresed in a 1% (*w*/*v*) agarose gel and stained with RedSafe Nucleic Acid Staining Solution (an alternative to ethidium bromide; iNtRON Biotechnology).

### 2.18. Statistical Analysis

Statistical analysis of data was performed using GraphPad Prism Software (version 7; San Diego, CA, USA). Significant differences in gene expression among experimental groups were determined by one-way analysis of variance followed by Tukey's multiple comparison test. Differences were considered significant at *p* < 0.05 (^∗^*p* < 0.05 and ^∗∗^*p* < 0.01 in figures). Data are presented as the mean ± standard error of the mean (S.E.M).

## 3. Results

### 3.1. Expression Analysis of the Dual-Fluorescence Reporter System in Porcine Embryos

**Figure 1:**
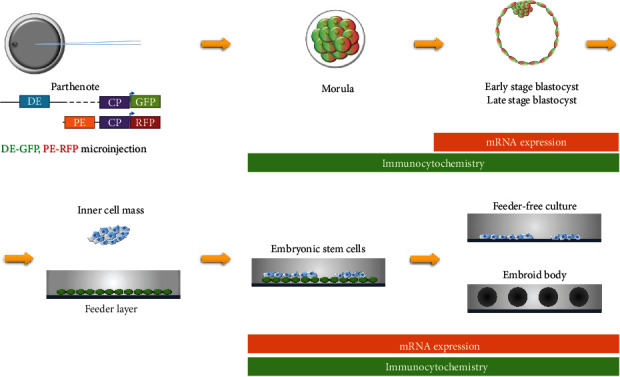
Schematic overview of the study on the porcine *OCT4* reporter system during early embryo development and embryonic stem cells. Porcine embryos were microinjected with the p*OCT4*-*Δ*PE-eGFP (DE-GFP) containing a distal enhancer and core promoter and p*OCT4*-*Δ*DE-DsRed2 (PE-RFP) containing a proximal enhancer and core promoter. Hatched parthenogenetic blastocysts were seeded on feeder cells composed of mitotically inactivated mouse embryonic fibroblasts (MEFs). Protein and mRNA expression in morula, blastocysts, embryonic stem cells, and embryoid body was analyzed using immunocytochemistry and quantitative real-time PCR.

**Figure 2:**
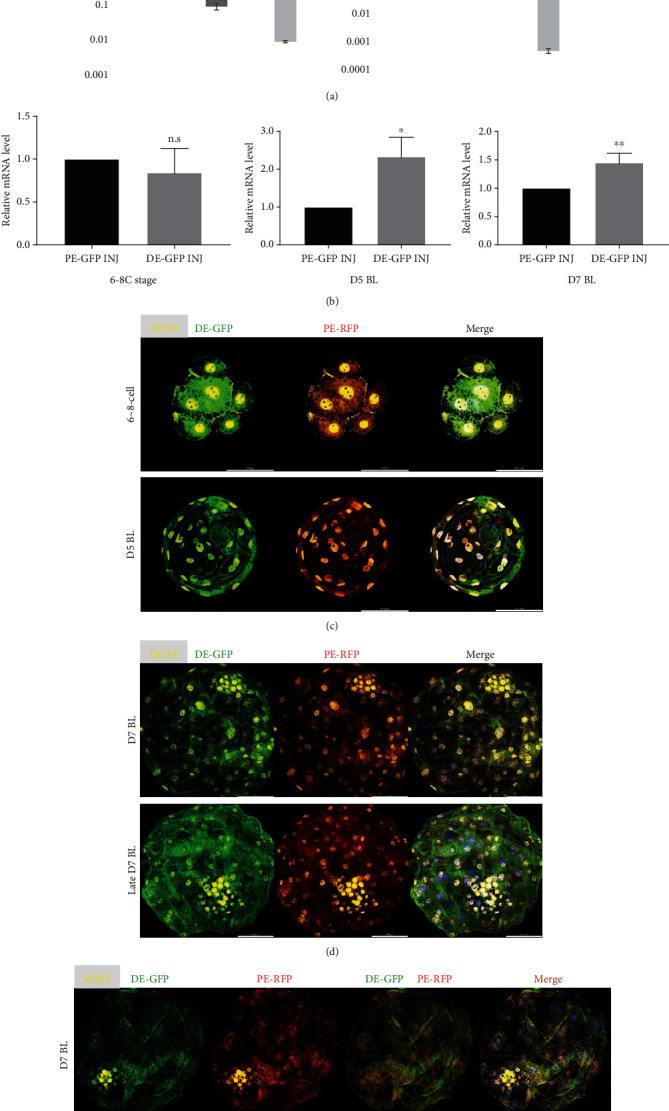
Expression of the *OCT4* reporter system during preimplantation porcine embryo development. (a) Expression levels of DE-GFP and PE-GFP were measured in 3 developmental stages (6–8 cells, early blastocyst and late blastocyst). Error bars represent the mean SEM. Values with different letters (a, b, and c) are significantly different. (b) Gene expression patterns of PE-GFP and DE-GFP in the *OCT4* reporter system-injected embryos. Each group has three replicates. ^∗^Significant differences. (^∗^*p* < 0.05, ^∗∗^*p* < 0.01). (c–e) Immunofluorescence analysis for PE-RFP, DE-GFP, pluripotent genes (OCT4 and SOX2–yellow), and DAPI nuclear staining in OCT4 reporter system-injected embryos. Size maker corresponds to 100 *μ*m.

In a recent study, the establishment of authentic porcine ESCs was reported (Choi et al. 2019) and it spurred the investigation of porcine pluripotency. However, naïve-state ESCs in pigs have not yet been reported and analysis of the activities of porcine *OCT4* enhancers could not use two different states of porcine stem cells. To overcome this problem, we introduced a dual-reporter system in porcine embryos and analyzed reporter system expression in naïve-state cells (Figure 1). Embryo production by parthenogenetic activation was used to reduce sperm-derived variables. The expression of DE-GFP and PE-GFP in the pooled embryos was highest at the 8-cell stage, and expression decreased through 5-day-old (D5, early) and 7-day-old (D7, late) blastocysts (BL) (Figure 2(a)). This is consistent with the highest expression of OCT4 at the 6–8-cell stage and decreases at later stages in preimplantation porcine embryos [34]. When comparing the expression of DE-GFP and PE-GFP, there was no difference in expression at the 6–8-cell stage, but at the D5 BL stage and D7 BL stage, the expression of DE-GFP was significantly increased compared with that of PE-GFP (Figure 2(b)). Next, to determine the dimensional gene expression pattern, the expression of DE-GFP and PE-RFP was inspected in porcine morula and D5 BL and D7 BL through immunocytochemistry assays. At the 6–8-cell stage, D5 BL stage, and D7 BL stage, PE-RFP and DE-GFP were expressed in most blastomeres along with OCT4 (Figure 2(c)). Even when the expression of OCT4 was reduced in TE, GFP and RFP were maintained (Figure 2(d)). To determine the difference in expression in ICM and TE of the reporter system, the preimplantation porcine ICM marker SOX2 was stained but there was no difference (Figure 2(e)). These results show that the two OCT4 enhancers in porcine naïve-state cells are both functional, although there is a difference in expression.

### 3.2. Characterization of Pig Embryonic Stem Cells with a Porcine OCT4 Reporter System

**Figure 3:**
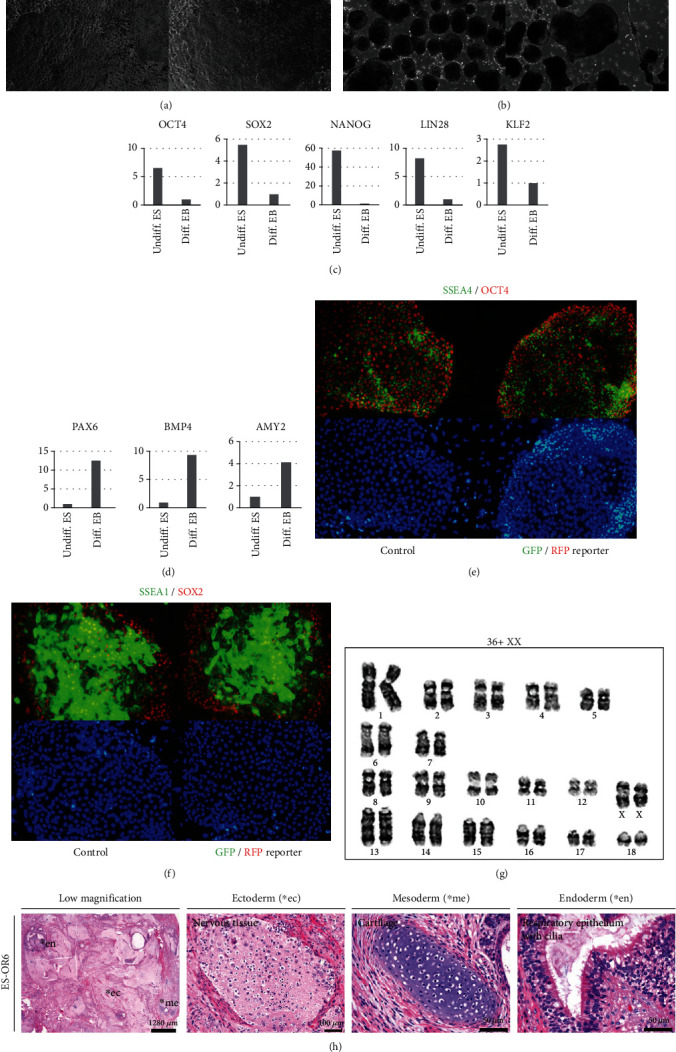
Characterization of porcine embryonic stem cells with the porcine *OCT4* reporter system. (a) Porcine embryonic stem cells with the *OCT4* reporter system. (b) Embryoid bodies aggregated from porcine embryonic stem cells. (c) Comparison of gene expression related to pluripotency between undifferentiated embryonic stem cells and differentiated embryoid bodies. (d) Comparison of gene expression related to differentiation between undifferentiated embryonic stem cells and differentiated embryoid bodies. (e) SSEA4 and OCT4. (f) SSEA1 and SOX2 in porcine embryonic stem cells with or without the *OCT4* reporter system. Pluripotency markers such as OCT4 and SOX2 and surface markers such as SSEA1 and SSEA4 coexpressed in pig embryonic stem cells with or without OCT4 reporter system. (g) Karyotyping of porcine embryonic stem cells with the OCT4 reporter system. Karyotyping showed that no chromosomal aberration occurred during in vitro culture. (h) Teratoma assay of porcine embryonic stem cells with the OCT4 reporter system. When injected subcutaneously into athymic nude mice, stem cells could generate into teratoma containing all three germ layers, such as nervous tissue, cartilage, and epithelium.

Pluripotent stem cells have two different features dependent on their pluripotent states, naïve and primed. Naïve pluripotent-state PSCs have a multilayer and dome-shaped morphology. Primed pluripotent-state PSCs have a single-layer and flattened morphology [1]. Porcine ESCs with a porcine-specific *OCT4* reporter system have single-layer and flattened morphology, and single cells have epithelial morphology with a high nucleus-to-cytoplasm ratio similar to that of human ESCs and mouse-primed epiblast stem cells (Figure 3(a)). Embryoid bodies from porcine embryonic stem cells with the porcine *OCT4* reporter system formed in suspension culture (Figure 3(b)). Porcine embryonic stem cells have higher expression of pluripotent genes such as *OCT4*, *SOX2*, *NANOG*, *LIN28*, and *KLF2* than embryoid bodies. Differentiation markers, including *PAX6*, *BMP4*, and *AMY2*, were normally expressed after differentiation of porcine embryonic stem cells with the porcine *OCT4* reporter system (Figures 3(c) and 3(d)). Porcine embryonic stem cells expressed pluripotent markers such as OCT4 and SOX2 and surface markers such as SSEA1 and SSEA4 (Figures 3(e) and 3(f)). Karyotyping showed that no chromosomal mutation occurred during in vitro culture (Figure 3(g)). When injected subcutaneously into BALB/c nude mice, stem cells could generate teratomas containing all three germ layers, such as hair follicles, cartilage, and epithelium (Figure 3(h)).

**Figure 4:**
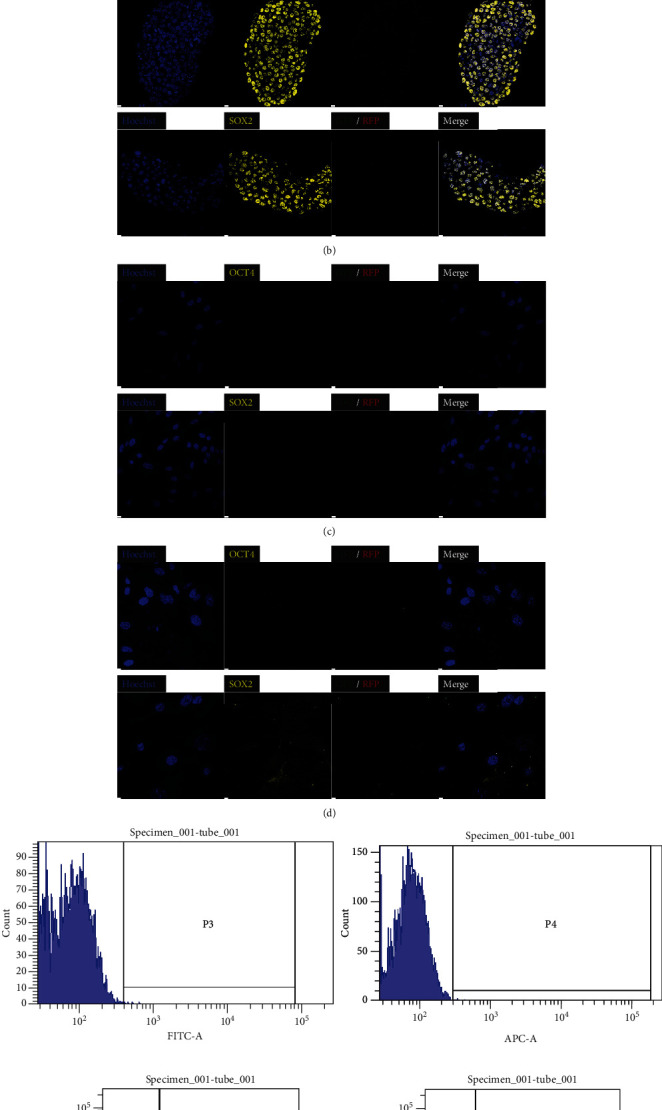
Expression of dual-porcine *OCT4* reporter system and pluripotency markers in porcine embryonic stem cells.Reporter fluorescent genes and pluripotency markers were checked in the protein expression level. (a) Porcine embryonic stem cells with the *OCT4* reporter system. (b) Porcine embryonic stem cells without the *OCT4* reporter system. (c) Porcine embryonic fibroblasts with the *OCT4* reporter system. (d) Embryonic bodies after differentiation from porcine embryonic stem cells with the *OCT4* reporter system. (e) Porcine embryonic stem cells without the reporter system. (f) Porcine embryonic stem cells with the reporter system. Flow cytometry showed that porcine embryonic stem cells with the *OCT4* reporter system have 99.6% positive rates of fluorescence compared to those of the control group.

**Figure 5:**
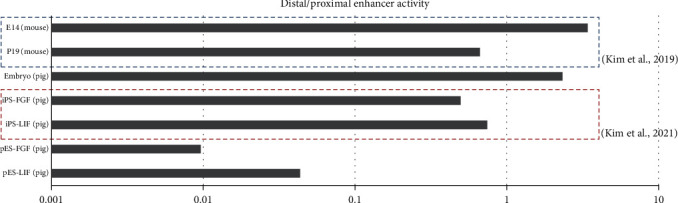
Comparison of the distal/proximal enhancer activity rate in pluripotent cells of mouse or pig. The activity of OCT4 distal and proximal enhancers was compared in various pluripotent cells. Cells with a naïve pluripotent state (mouse E14 ESCs, porcine embryo) had a value greater than 1, which indicates higher activity of the distal enhancer than the proximal enhancer. On the other hand, cells known as the primed pluripotent state (mouse P19 ECs, FGF- or LIF-dependent piPSCs) had a value less than 1, which indicated that the proximal enhancer has higher activity than the distal enhancer. E14 mouse embryonic stem cells, P19 mouse embryonic carcinoma, porcine embryo, FGF- or LIF-dependent porcine induced pluripotent stem cells, and porcine embryonic stem cells with FGF- or LIF-supplemented culture media. Mouse E14 and P19 cells were analyzed by using luciferase assay in (Kim et al., 2019), and porcine-induced pluripotent stem cells were analyzed in (Kim et al., 2021).

**Figure 6:**
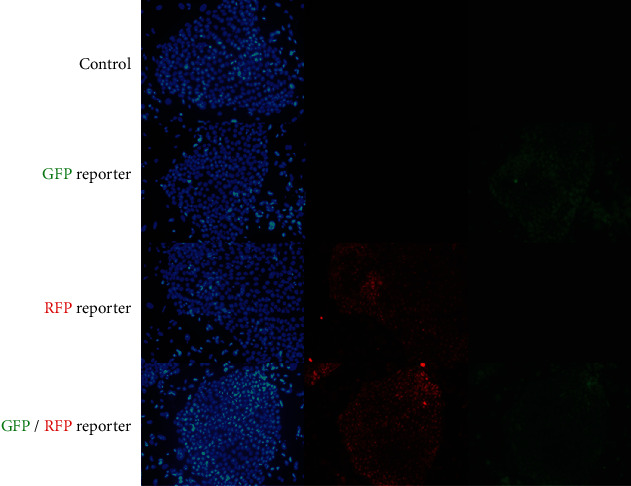
Expression of the fluorescent signal in porcine embryonic stem cells using the single-color or dual-reporter system. The porcine *OCT4* reporter system consists of two reporters: DE-GFP and PE-RFP. Because each reporter is separate from the other, the reporter system can be used simultaneously. Control: porcine embryonic stem cells without any *OCT4* reporter system. GFP reporter: porcine embryonic stem cells with the DE-GFP reporter vector only. RFP reporter: porcine embryonic stem cells with the PE-RFP reporter vector only. GFP/RFP reporter: porcine embryonic stem cells with the dual-*OCT4* reporter system.

**Figure 7:**
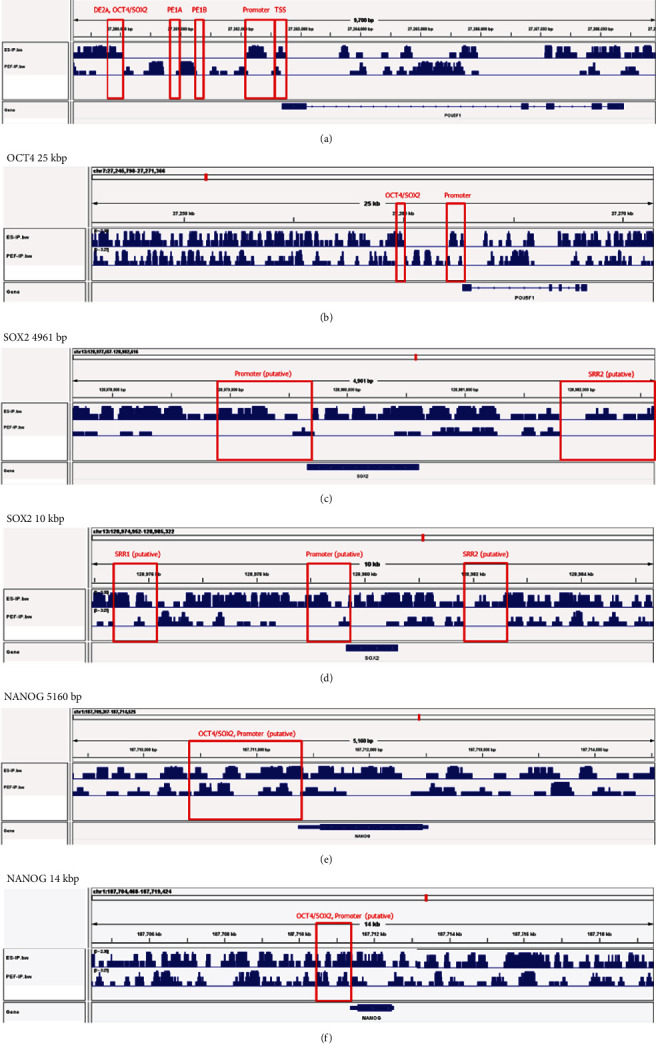
Genomic snapshots of porcine *OCT4* ChIP-seq nearby pluripotent markers *OCT4*, *SOX2*, and *NANOG* in porcine embryonic stem cells and embryonic fibroblasts. (a) 9700 bp sequence around *OCT4*. (b) 25 kbp sequence around *OCT4*. (c) 4961 bp sequence around *SOX2*. (d) 10 kbp sequence around *SOX2*. (e) 5160 bp sequence around *NANOG*. (f) 14 kbp sequence around *NANOG*.

The expression of GFP and RFP, which are produced by distal or proximal enhancers, respectively, and pluripotent markers such as *OCT4*, *SOX2*, and *NANOG* in pig ESCs with a porcine *OCT4* reporter system was examined by immunostaining (Figure 4(a)). On the other hand, porcine embryonic stem cells without a porcine *OCT4* reporter system expressed only pluripotent marker genes (Figure 4(b)). Porcine embryonic fibroblasts with a porcine *OCT4* reporter system were not pluripotent stem cells. Therefore, they did not express any pluripotent marker genes or fluorescent proteins produced by the reporter system (Figure 4(c)). After differentiation, embryonic bodies aggregate from porcine embryonic stem cells with a porcine *OCT4* reporter system and lose their pluripotency after differentiation. They did not express fluorescent proteins and only expressed SOX2, which is a pluripotent marker and neural differentiation marker (Figure 4(d)). Flow cytometry showed that porcine embryonic stem cells with the OCT4 reporter system had 99.6% positive rates of fluorescence compared to the control group (Figures 4(e) and 4(f) and Figure S3). The activity of *OCT4* distal and proximal enhancers was compared in various pluripotent cells. Cells in the naïve pluripotent state (mouse E14 ESCs, porcine embryo) had a value greater than 1, which indicated that the distal enhancer has higher activity than the proximal enhancer. On the other hand, cells known to exist in a primed pluripotent state (mouse P19 ECs, FGF-, or LIF-dependent piPSCs) had a value less than 1, which indicated that the proximal enhancer has higher activity than the distal enhancer. E14 mouse embryonic stem cells and porcine embryos are well known as naïve pluripotent cells. The rates of activity of *OCT4* distal and proximal enhancers in mouse embryonic stem cells and porcine embryos confirmed that they are present in naïve pluripotent states. On the other hand, P19 mouse embryonic carcinoma is one of the cell types representing a primed pluripotent state. Analysis using *OCT4* upstream regulatory region-based reporters showed that primed pluripotent-state cells use proximal enhancers more than distal enhancers to produce *OCT4*. Based on these results, pig embryonic fibroblasts with a reporter system were found to be close to the primed pluripotent state under FGF- or LIF-dependent reprogramming conditions. In the same way, porcine embryonic stem cells with the reporter system were also found to be close to the primed state. To try to convert the pluripotent state (primed to naïve) of embryonic stem cells, they were cultured under LIF and WH-4-023 conditions. Porcine embryonic stem cells with LIF, WH-4-023, ActA, IWR, and CHIR had different morphological features from their counterparts with FGF, ActA, IWR, and CHIR. The ratio of distal enhancer activity divided by proximal enhancer activity in porcine embryonic stem cells with LIF was higher than that in porcine embryonic stem cells with FGF, but it was still close to the primed pluripotent state (Figure 5). The porcine *OCT4* reporter system consists of two reporters: DE-GFP and PE-RFP. If necessary, dual reporters can be used simultaneously or separately (Figure 6). When we examined ChIP-seq signals at pluripotent markers such as *OCT4*, *SOX2*, and *NANOG* sites in porcine embryonic stem cells using integrated genomics viewer (IGV) 2.9.4, we observed peaks at porcine *OCT4* DE 2A and OCT4/SOX2 binding sites (DE 2B), the *SOX2* putative promoter and enhancers (SRR1, SRR2), and the *NANOG* putative promoter region. Additionally, peaks were found in regions containing promoter and transcription start sites. There was no signal at the *OCT4* proximal enhancer elements (Figure 7, Table S3, Figures S1 and S2).

## 4. Discussion

In a recent study, the establishment of authentic porcine ESCs was reported [8] and it spurred the investigation of porcine pluripotency. However, naïve-state ESCs in pigs have not yet been reported and analysis of the activities of porcine *OCT4* enhancers could not use two different states of porcine stem cells. To overcome this problem, we conducted a sequence analysis of the porcine *OCT4* upstream region and suggested some reporter systems [32] and introduced a dual-reporter system in mouse embryonic stem cells, mouse carcinoma, and porcine-induced pluripotent stem cells. The results indicated porcine *OCT4* enhancers, and the reporter system were more similar to humans than to mice [32, 35]. In addition, we recently reported pig embryonic stem cells with an *OCT4* reporter system [36]. In this study, we introduced the reporter system into porcine embryos and embryonic stem cells and analyzed the expression of fluorescent produced the reporter in these cells. The results of this study showed that the activity of the *OCT4* enhancers differs from species to species. The distal enhancer was used only in preimplantation blastocysts in mouse [19], whereas the proximal enhancer and distal enhancer were used in most blastocyst cells in pig (Figures 2(d) and 2(e)). We confirmed the establishment of pig embryonic stem cells containing the *OCT4* reporter system (Figures 3 and 4), but the porcine embryonic stem cells were in primed states (Figure 5).

ChIP stands for chromatin immunoprecipitation, which aims at identifying DNA sequences binding to specific proteins as an important tool for identifying interactions between proteins and DNA sequences in cells. This is often used to study the binding sites and mechanism of transcription factors that regulate gene expression. Genome-wide profiling of porcine OCT4 ChIP-seq results indicated that porcine OCT4 is only involved in the distal enhancer region, which has the OCT4/SOX2 binding site, except for the proximal enhancer region in porcine embryonic stem cells. It is also involved in the porcine *OCT4* promoter region and transcription start sites. These results support the analysis of the upstream regulatory region of porcine *OCT4* obtained from the sequence analysis. *OCT4* plays an important role in establishing and maintaining the pluripotency network in concert with *SOX2* [37]. *OCT4* and *SOX2* are required in embryo development and pluripotent stem cells, and these genes are controlled in embryos and stem cells. These genes work together closely, and they have a negative feedback loop balance [38]. Peaks of porcine OCT4 ChIP-seq are identified at putative SOX2 promoters and enhancers (SRR1 and SRR2), so crossvalidation is also required in ChIP-seq of porcine SOX2 and OCT4. In addition, an assay for transposase-accessible chromatin using sequencing (ATAC-seq) is required for chromatin accessibility of the DE and PE in the porcine *OCT4* upstream region. In the somatic cell reprogramming process, epigenetic changes and the binding of the OCT4 gene were reported. H3K4me1 and H3K27ac are associated with enhancers, while H3K27me3 and H3K4me3 are associated with the promoter [39]. Not only transcription factor ChIP-seq of pluripotent markers such as OCT4 or SOX2 but also histone modification ChIP-seq including H3K4me1, H3K27ac, H3K27me3, and H3K4me3 are important for identifying the activities of the DE and PE.

## 5. Conclusions

We revealed the function of the species-specific *OCT4* reporter system and the early developmental *OCT4* enhancer pattern in porcine preimplantation embryos. Furthermore, porcine embryonic stem cells with a porcine-specific reporter system could be established and functioned in previously reported chemically defined medium. The ESCs with the reporter system expressed GFP and RFP, which are normally produced by *OCT4* enhancers and pluripotent genes such as *OCT4*, *SOX2*, and *NANOG*. This study confirmed that porcine embryonic stem cells in FGF2, activin A, and Wnt activator, which could support the maintenance of porcine ESCs with feeder cells, were in a primed pluripotent state. ChIP-seq data enabled genome-wide profiling of porcine *OCT4* in pig embryonic stem cells. The data support the results of the analysis of the upstream regulatory region of porcine *OCT4* obtained from the sequence analysis and help identify a candidate group of marker genes involved in porcine-specific pluripotency. Finally, studies on embryonic pluripotent cells with a porcine *OCT4* reporter system will help to improve the understanding of species-specific pluripotency and aid in improving human welfare with respect to healthy life and agricultural production.

## Data Availability

The datasets used in the current study are available from the corresponding author upon reasonable request by email.

## Supplementary Materials

Supplementary Materials Supplementary Table 1: oligo sequences of primers used in this paper. Supplementary Table 2: antibody list used in this paper. Supplementary Table 3: stats of porcine OCT4 ChIP-seq in porcine embryonic stem cells and porcine embryonic fibroblasts. Supplementary Figure 1: quality scores across all bases (per base quality) of IP and total input samples of porcine embryonic stem cells and embryonic fibroblasts. Supplementary Figure 2: statistical analysis of porcine OCT4 ChIP-seq in porcine embryonic stem cells and embryonic fibroblasts. Supplementary Figure 3: flow cytometry of porcine embryonic stem cells with or without the OCT4 reporter system.
