# Supplementary material for: Species-Specific Enhancer Activity of OCT4 in Porcine Pluripotency: The Porcine OCT4 Reporter System Could Monitor Pluripotency in Porcine Embryo Development and Embryonic Stem Cells
Source: Stem Cells Int. 2022 Jun 11;2022:6337532. doi: 10.1155/2022/6337532 (PMC9277468; doi:10.1155/2022/6337532)
Supplement: Supplementary Materials — Supplementary Table 1: oligo sequences of primers used in this paper. Supplementary Table 2: antibody list used in this paper. Supplementary Table 3: stats of porcine OCT4 ChIP-seq in porcine embryonic stem cells and porcine embryonic fibroblasts. Supplementary Figure 1: quality scores across all bases (per base quality) of IP and total input samples of porcine embryonic stem cells and embryonic fibroblasts. Supplementary Figure 2: statistical analysis of porcine OCT4 ChIP-seq in porcine embryonic stem cells and embryonic fibroblasts. Supplementary Figure 3: flow cytometry of porcine embryonic stem cells with or without the OCT4 reporter system. [file 6337532.f1.docx]

| Primary Antibodies | Target | Host | Company | Catalog Number |
| --- | --- | --- | --- | --- |
|  | SOX2 | Rabbit | Millipore | AB5603 |
|  | OCT4 | Rabbit | Santa Cruz | sc-9081 |
|  | NANOG | Rabbit | Peprotech | 500-P236 |
|  | RFP | Mouse | Invitrogen | MA5-15257 |
|  | GFP | Chicken | Invitrogen | A-10262 |
| Secondary Antibodies | Fluorescent dye | Target/Host | Company | Catalog Number |
|  | Alexa594 | Rabbit/Goat | Invitrogen | A-11012 |
|  | Alexa594 | Rabbit/Donkey | Invitrogen | A-21207 |
|  | Alexa488 | Chicken/Goat | Invitrogen | A11039 |
|  | Alexa555 | Rabbit/Goat | Invitrogen | A-21428 |
|  | Alexa647 | Mouse/Goat | Invitrogen | A332728 |

**Supplementary Table 1. List of antibodies.**

| GFP | F | GCGACGTAAACGGCCACAAGTTC |
| --- | --- | --- |
|  | R | GACCATGTGATCGCGCTTCTCG |
| RFP | F | TGAAGGGCGAGACCCACAA |
|  | R | TGGACTTGAACTCCACCAGGTA |
| OCT4 | F | CTTGGAGAGCCCTGGTTTTACT |
|  | R | GCCAGGTCCGAGGATCAAC |
| SOX2 | F | CGGCGGTGGCAACTCTAC |
|  | R | TCGGGACCACACCATGAAAG |
| NANOG | F | CATCTGCTGAGACCCTCGAC |
|  | R | GGGTCTGCGAGAACACAGTT |
| LIN28 | F | AAACGCAGATCCAAAGGAGA |
|  | R | GCTCAATTCTGAGCCTCTGG |
| KLF2 | F | TTCGGTATCTTTGACGACCCG |
|  | R | GGCTTGGCCTCTAGTAGCTC |
| PAX6 | F | AGAGAAGACAGGCCAGCAAC |
|  | R | GGCAGAGCACTGTAGGTGTT |
| BMP4 | F | CGTTGGTCTCGAGTATCCCG |
|  | R | AGAGTTTTCGCTGGTCCCTG |
| AMY2 | F | TGCTCTTGAATGTGAGCGGT |
|  | R | TACGGACGCCAACGTTGTTA |
| GAPDH | F | TGCTCCTCCCCGTTCGAC |
|  | R | ATGCGGCCAAATCCGTTC |

**Supplementary Table 2. List of primers.**

**Supplementary Table 3. Stats of porcine OCT4 ChIP-seq in porcine embryonic stem cells and porcine embryonic fibroblasts.**

The total number of bases, reads, GC (%), Q20 (%), and Q30 (%) were calculated. Total read bases is the total number of bases sequenced. It is calculated as total reads x read length. Total reads indicated total number of reads. GC (%): GC content, Q20 (%): Ratio of bases that have Phred quality scores greater than or equal to 20, Q30 (%): Ratio of bases that have Phred quality scores greater than or equal to 30. Processed reads: Number of cleaned reads after trimming. Mapped reads: Number of reads mapped to reference. Read that failed to align: Number of reads that failed to align. Suppressed multiple mapped reads: Number of reads removed due to multiple mapping.


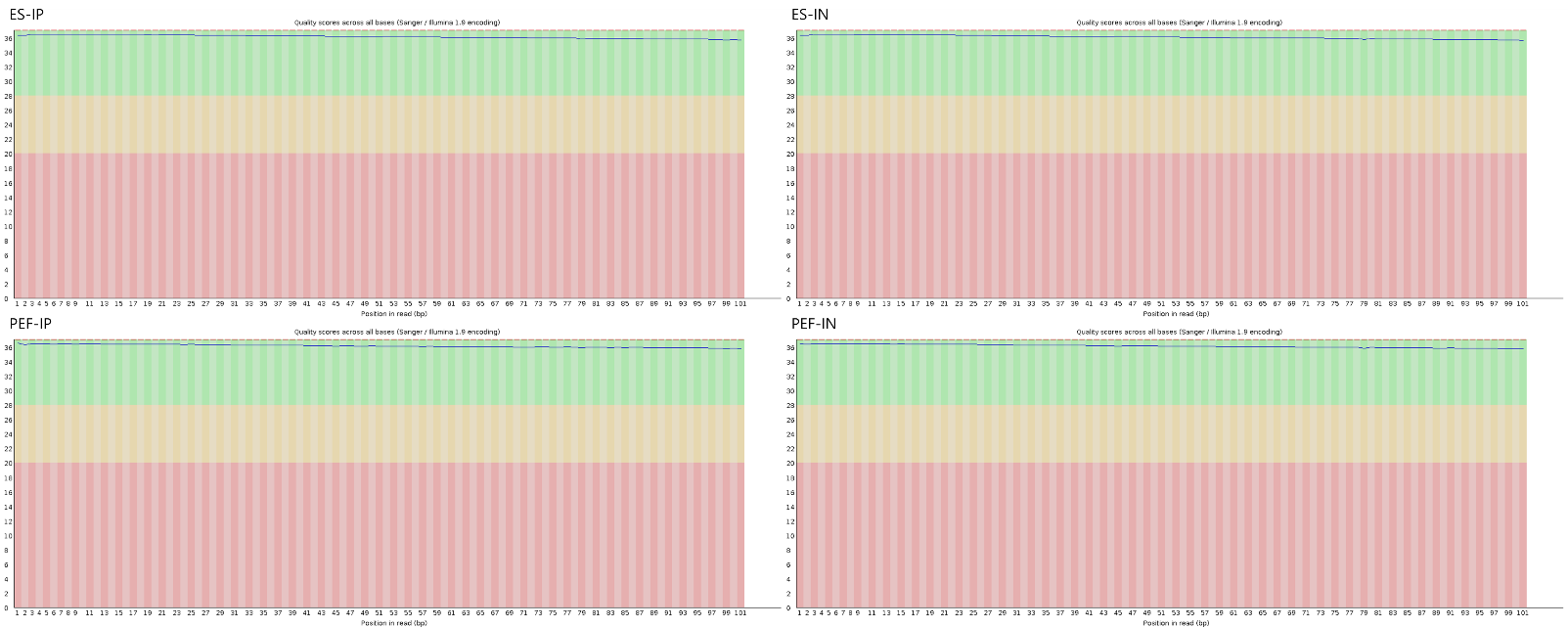


**Supplementary Figure 1. Quality scores across all bases (per base quality) of IP and total input samples of porcine embryonic stem cells and embryonic fibroblasts.**

The quality of the produced data is determined by the phred quality score at each cycle. A box plot containing the average quality at each cycle was created with FastQC. The x-axis shows the number of cycles, and the y-axis shows the Phred quality score. A Phred quality score of 20 means 99% accuracy, and reads over a score of 20 are accepted as good quality. Yellow box: Interquartile range (25-75%) of phred score at each cycle, Red line: Median of phred score at each cycle, Blue line: Average of phred score at each cycle, Green background: Good quality, Orange background: Acceptable quality, Red background: Bad quality.


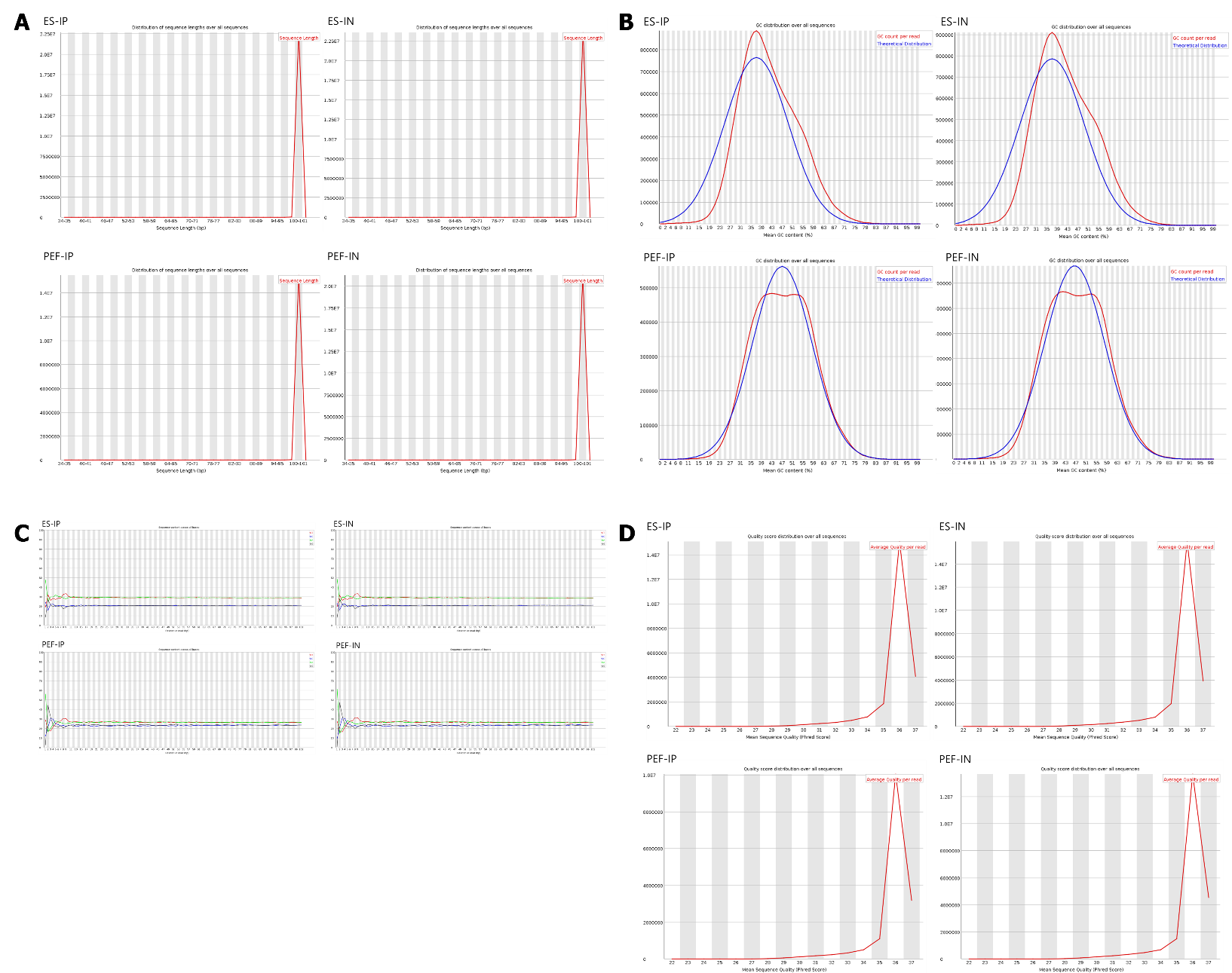


**Supplementary Figure 2. Statistical analysis of porcine OCT4 ChIP-seq in porcine embryonic stem cells and embryonic fibroblasts.**

A. Distribution of sequence lengths over all sequences (sequence length distribution) of IP and total input samples of porcine embryonic stem cells and embryonic fibroblasts. B. GC distribution over all sequences (per sequence GC content) of IP and total input samples of porcine embryonic stem cells and embryonic fibroblasts. C. Sequence content across all bases (per base sequence content) of IP and total input samples of porcine embryonic stem cells and embryonic fibroblasts. D. Quality score distribution over all sequences (per sequence quality) of IP and total input samples of porcine embryonic stem cells and embryonic fibroblasts.

**
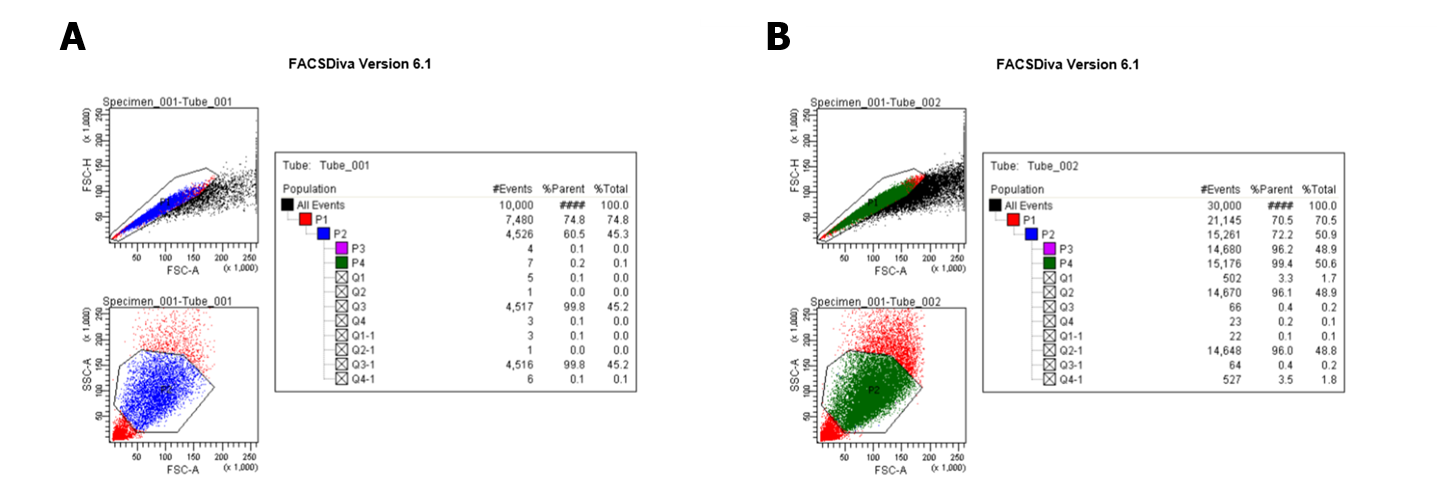
Supplementary Figure 3. Flow cytometry of porcine embryonic stem cells with or without the *OCT4* reporter system.**

A. The control group consisted of porcine embryonic stem cells without the *OCT4* reporter system, and B. The experimental group consisted of porcine embryonic stem cells with the *OCT4* reporter system.
